# Cr(VI) reduction and physiological toxicity are impacted by resource ratio in *Desulfovibrio vulgaris*

**DOI:** 10.1007/s00253-017-8724-4

**Published:** 2018-02-10

**Authors:** Lauren C. Franco, Sadie Steinbeisser, Grant M. Zane, Judy D. Wall, Matthew W. Fields

**Affiliations:** 1 Department of Microbiology and Immunology, Montana State University, Bozeman, MT USA; 2 Center for Biofilm Engineering, Montana State University, 366 Barnard Hall, Bozeman, MT 59717 USA; 3 Departments of Biochemistry and Molecular Microbiology and Immunology, University of Missouri-Columbia, Columbia, MO USA; 4 ENIGMA, Berkeley, CA, USA, http://enigma.lbl.gov

**Keywords:** Chromate, Sulfate-reducing bacteria, Energy balance

## Abstract

*Desulfovibrio* spp. are capable of heavy metal reduction and are well-studied systems for understanding metal fate and transport in anaerobic environments. *Desulfovibrio vulgaris* Hildenborough was grown under environmentally relevant conditions (i.e., temperature, nutrient limitation) to elucidate the impacts on Cr(VI) reduction on cellular physiology. Growth at 20 °C was slower than 30 °C and the presence of 50 μM Cr(VI) caused extended lag times for all conditions, but once growth resumed the growth rate was similar to that without Cr(VI). Cr(VI) reduction rates were greatly diminished at 20 °C for both 50 and 100 μM Cr(VI), particularly for the electron acceptor limited (EAL) condition in which Cr(VI) reduction was much slower, the growth lag much longer (200 h), and viability decreased compared to balanced (BAL) and electron donor limited (EDL) conditions. When sulfate levels were increased in the presence of Cr(VI), cellular responses improved via a shorter lag time to growth. Similar results were observed between the different resource (donor/acceptor) ratio conditions when the sulfate levels were normalized (10 mM), and these results indicated that resource ratio (donor/acceptor) impacted *D. vulgaris* response to Cr(VI) and not merely sulfate limitation. The results suggest that temperature and resource ratios greatly impacted the extent of Cr(VI) toxicity, Cr(VI) reduction, and the subsequent cellular health via Cr(VI) influx and overall metabolic rate. The results also emphasized the need to perform experiments at lower temperatures with nutrient limitation to make accurate predictions of heavy metal reduction rates as well as physiological states in the environment.

**Electronic supplementary material:**

The online version of this article (10.1007/s00253-017-8724-4) contains supplementary material, which is available to authorized users.

## Introduction

Chromium is a naturally occurring trace element that is widely used for industrial purposes (i.e., metallurgy, leather tanning) and has become a major pollutant of soil and groundwater (Barnhart 1997; Zayed and Terry 2003; Dhal et al. 2013). Hexavalent chromium is soluble and can easily be transported across cell membranes whereas trivalent chromium is mostly insoluble and therefore not as toxic to cells. Cr(VI) is a known mutagen and carcinogen, although it appears that chromium toxicity is largely caused by reactive intermediates such as Cr(V) or hydroxyl radicals produced during Cr(VI) reduction and Cr(III) forming adducts with DNA or natural organic matter (Nriagu and Nieboer 1988; Aiyar et al. 1991; Zhitkovich et al. 1995; 2013; Gustafsson et al. 2014).

*Desulfovibrio* species are model sulfate-reducing bacteria (SRB) and have been shown to reduce metals, metalloids, and radionuclides (Heidelberg et al. 2004; Klonowska et al. 2008; Zhou et al. 2014). Previous work with purified enzymes and whole cells has shown that *Desulfovibrio vulgaris* is capable of reducing Cr(VI) via hydrogenases and cytochrome c3, but cells are unable to use Cr(VI) as a terminal electron acceptor linked to growth (Lovley and Phillips 1994; Chardin et al. 2002; Elias et al. 2004). One promising approach for treatment of contaminated environments is in situ biostimulation, the process of promoting indigenous microbial activity via the addition of carbon, nitrogen, phosphorus, and/or energy sources (Tyagi et al. 2011). Typically, microbial activity is promoted through the addition of carbon/energy sources as soluble substrates and/or more recalcitrant, complex substrates that degrade more slowly (Faybishenko et al. 2008). For example, biostimulation has been used successfully to reduce contaminants at the Cr(VI) contaminated 100-H area of the Hanford Site in south-central Washington state where Cr(VI) levels can be up to 50 ppm (Faybishenko et al. 2008). Injections of electron donors such as polylactate hydrogen-release compound (HRC) into the subsurface have been shown to stimulate Cr(VI)-reducing microorganisms such as *Desulfovibrio* spp. and other metal-reducing bacteria in situ (Zhang et al. 2015).

Electron donors and electron acceptors are rarely balanced according to metabolic stoichiometry for a particular organism or guild. In fact, it is common practice to stimulate growth of existing microorganisms by injecting an excess electron donor into the subsurface, creating an electron acceptor limited environment. Previous studies have reported that substrate limitation can increase Cr(VI) susceptibility (Chardin et al. 2002), and injections of electron donor without a corresponding increase in electron acceptor creates unbalanced electron donor to acceptor ratios. However, little is known about the physiological responses of metal-reducing populations in the context of unbalanced ratios that occur as a consequence of stimulated conditions. In addition, metal-reducing bacteria are often studied at optimal growth temperatures (between 30 and 40 °C) although much lower temperatures are experienced in situ. A previous study examined uranium reduction in a sulfate-reducing consortium at 10, 20, and 30 °C, and observed that growth and reduction kinetics were affected by temperature (Boonchayaanant et al. 2008). Therefore, it is important to understand the growth characteristics of microorganisms that are capable of heavy metal immobilization, such as SRB, under typical field conditions to accurately assess and predictability and rate of contaminant immobilization. To our knowledge, previous studies have not evaluated Cr(VI) effects on SRB growth and reduction rates at a temperature lower than 30 °C under unbalanced stoichiometries of electron donors to acceptors (resource ratio).

## Materials and methods

### Strain/growth conditions

The *D. vulgaris* Hildenborough culture, deposited as ATCC29579, was acquired from Dr. Romy Chakraborty (Lawrence Berkeley National Laboratory). LS4D medium (Borglin et al. 2009) (modified to 2.5 μM resazurin and 130 μM riboflavin in Thauer’s vitamins (Brandis and Thauer 1981)) was prepared anoxically by boiling water under oxygen-free N_2_ gas, adding medium components, and dispensing into N_2_ gassed tubes or serum bottles sealed with butyl stoppers and aluminum crimp seals as previously described (Klonowska et al. 2008). LS4D medium was modified to alter electron donor (lactate) to electron acceptor (sulfate) ratios, 60 to 30 mM (balanced, or 20:10), 50 to 10 mM (electron acceptor-limited), and 18 to 50 mM (electron donor-limited or 5:10). The medium was not prepared with a reducing agent to avoid abiotic Cr(VI) reduction. Inocula for all experiments unless otherwise noted were grown in the same electron donor to acceptor ratio (i.e., inoculum grown in balanced medium was inoculated into balanced medium). Cultures were grown at ambient room temperature (approximately 20 °C) unless otherwise stated.

Inocula for all experiments were prepared by washing mid-exponential phase (approximately 0.4 OD_600_) cultures to remove sulfide as previously described (Klonowska et al. 2008). To do this, cultures (9 ml aliquots) were harvested by centrifugation at 5750×*g* for 10 min at room temperature. The supernatant was removed anoxically and aseptically by needle attached to a vacuum flask under constant flow of N_2_ gas to maintain neutral pressure. Pellets were then re-suspended in 9 ml fresh LS4D medium that contained lactate and sulfate concentrations of the respective growth condition and washed once more, after which the pellets were re-suspended and concentrated in 1 ml fresh LS4D. Concentrated cell cultures were inoculated into 25 ml balch tubes with LS4D medium (15 ml) that contained Cr(VI) (potassium chromate) at 0, 50, and 100 μM to an OD_600_ between 0.06 and 0.07. Growth was monitored by optical density (600 nm) and samples of 0.2 to 0.5 ml were withdrawn throughout the growth cycle to monitor Cr(VI), lactate, acetate, and sulfate concentrations. All experiments were performed in triplicate. Ascorbate, which has been shown to reduce Cr(VI) to Cr(III) and form short-lived Cr(V) and Cr(IV) intermediates and Cr(III)-ascorbate complexes (Cieǎṡlak-Golonka et al. 1992; Stearns and Wetterhahn 1994), was added to cultures with and without Cr(VI) to determine the effect that complexed Cr(III) had on growth. Cells were prepared as described above and inoculated into electron acceptor-limited LS4D medium that contained either 50 μM sodium ascorbate, 50 μM potassium chromate, 50 μM sodium ascorbate and 50 μM potassium chromate, or 50 μM potassium chromate added prior to inoculation and 50 μM sodium ascorbate added 3 h post-inoculation.

### Experimental design

In order to determine the physiological responses and Cr(VI) reduction rates under field relevant conditions, *D. vulgaris* was grown under three conditions of resource ratios with and without Cr(VI) (50 and 100 μM) at 20 °C. A ratio of 60:30 (or 20:10) mM (lactate/sulfate) was used for balanced (BAL), 50:10 mM for electron-acceptor limited (EAL), and 18:50 (5:10) mM for electron-donor limited (EDL). Cell cultures were washed of sulfides to observe cellular responses to field relevant levels of Cr(VI). Cr(VI) reduction rates, growth rates, biomass yields (*Y*^sulfate^), and viability were measured to assess physiological responses. Similar levels of lactate (electron-donor) were tested with increasing levels of sulfate (electron-acceptor), and protection with ascorbate as well as growth in a sulfate permease mutant showed the role of Cr(VI) influx, metabolism, and toxicity on cell viability and Cr(VI) reduction.

### Cr(VI) reduction analysis

Cr(VI) reduction was measured by quantifying Cr(VI) concentrations over time using the diphenylcarbazide method with Hach ChromaVer 3 reagent (Hach Company, Loveland, CO) as previously described (Viamajala et al. 2002; Klonowska et al. 2008) except that absorbance was read in disposable cuvettes (Plastibrand, Germany) at 540 nm. Cells were removed by filtration (0.22 μm) so that only Cr(VI) in the filtrate was measured. Primary and secondary Cr(VI) reduction rates were calculated from bi-phasic decline Cr(VI) levels and the culture density did not change significantly from inoculation during the time in which the rates were calculated. Cr(VI) in uninoculated medium and cultures containing heat-killed cells were also measured.

### Analytical techniques

Lactate and acetate concentrations were measured by HPLC (Dionex) with an Aminex HPX-87H ion exclusion column. Sulfate concentrations were measured by ion chromatography (Dionex) with an IonPac AS11 column (Dionex). Cell viability was measured via the most probable number (MPN) method at 3 and 48 h. Washed cells were inoculated into fresh LS4D medium (~ 0.07 OD_600_) that was electron donor to acceptor balanced or electron acceptor-limited and contained 0 or 50 μM K_2_CrO_4_. Subsamples were removed and serially diluted with LS4D medium that contained sodium sulfide as a reducing agent. MPN cultures were incubated at 30 °C for 3 weeks at which point the MPN for each condition was calculated (Jarvis et al. 2010).

### Mutant generation

Construction of the mutants lacking the annotated sulfate permease genes, *sulP*, was accomplished similarly to the generation of marker-less deletion strains in the uracil phosphoribosyltransferase (*upp*, pyrimidine salvage pathway enzyme) deletion mutant (JW710; resistant to 5-fluoro uracil (5FU)) of *Desulfovibrio vulgaris* Hildenborough (Keller et al. 2009). In short, two plasmids were necessary for each gene deletion. The first (marker-exchange) plasmid contained the antibiotic-resistance cassette encoding the aminoglycoside-3′-phosphotransferase (*npt*, Km^r^) driven by the native promoter (P_*npt*_) followed by the *upp* gene (P_*npt*_*-npt-upp*) and was exchanged with the gene of interest (selected with the kanamycin analog G418). The second (marker-less deletion) plasmid was used for removing the cassette that conferred sensitivity to 5FU (selected with 5FU) resulting in the in-frame, marker-less deletion. Following each transformation, putative transformants were screened for phenotypes with spectinomycin, 5FU, and G418 to determine the probability that a double homologous recombination event took place rather than a single recombination event resulting in plasmid insertion. Three of the isolates with correct phenotypes were further confirmed by Southern blot analysis and one selected as the confirmed deletion strain. Multiple deletions were obtained through an iterative process of transforming the appropriate parental strain with the marker-exchange/marker-less deletion plasmids and selecting for the appropriate resistances. A total of nine plasmids were constructed for generating the deletion strains of the three putative sulfate-permease genes (Table S1). Each marker-exchange plasmid was initially constructed with only the *npt* gene and no *upp* gene, as done in other studies (Parks et al. 2013; Lovley and Phillips 1994; Li and Elledge 2007). These plasmids were later modified to include the *upp* gene as the second gene in an artificial operon driven by P_*npt*_. This was accomplished by PCR amplifying the plasmid with a pair of primers that allowed for the addition of the counter-selectable feature by the SLIC (sequence- and ligation- independent cloning) technique (Li and Elledge 2007). The construct for a marker-less, in-frame deletion plasmid was produced by PCR amplification of the original marker-exchange plasmid with primers that excluded the kanamycin-resistance marker and ligated closed using the SLIC procedure. The regions of each plasmid necessary for homologous recombination were sequenced (DNA core, University of Missouri, Columbia) to determine fidelity to the published sequence. These plasmids were transformed into JW710 for the single deletion strains or into one of the marker-less deletion strains to generate strains deleted of multiple genes. All primers are listed in Table S2.

## Results

### Temperature affects *D. vulgaris* growth and Cr(VI) reduction/toxicity under a balanced resource ratio

**Fig. 1:**
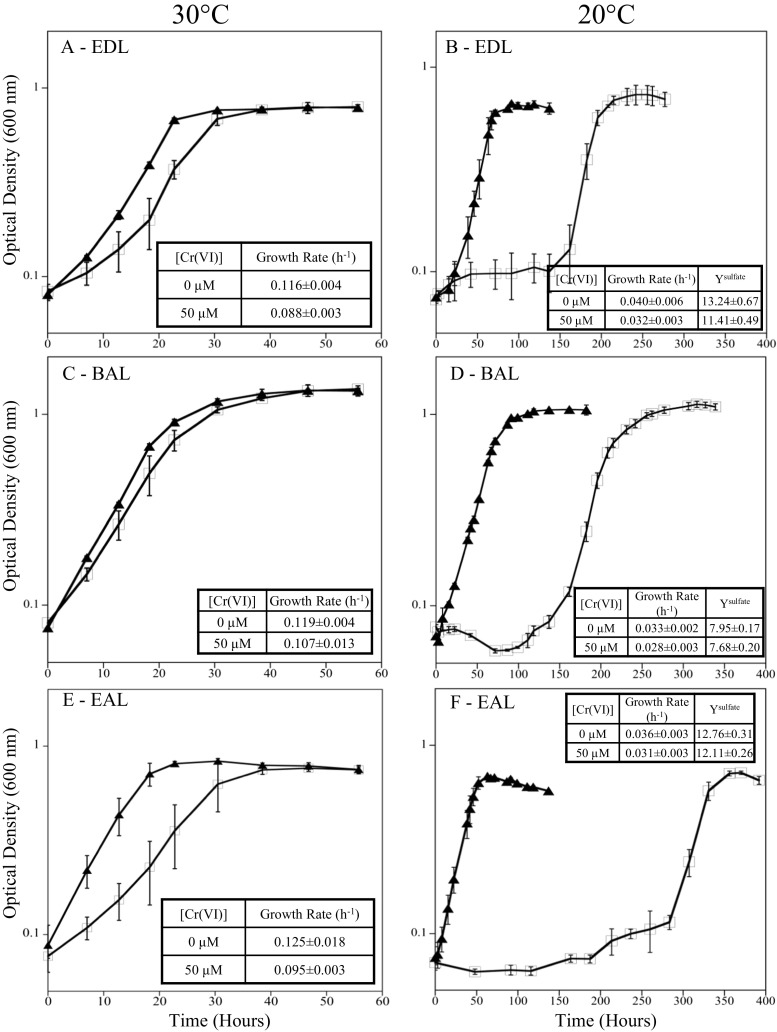
Growth of *D. vulgaris* at 30 °C (**a**, **c**, and **e**) and 20 °C (**b**, **d**, and **f**) under EDL (**a**, **b**), BAL (**c**, **d**), and EAL (**e**, **f**) conditions with 0 (●) and 50 (◻) μM Cr(VI). Growth rates (h^−1^) and growth yields (grams of protein/moles sulfate consumed) are overlaid for each condition

**Fig. 2:**
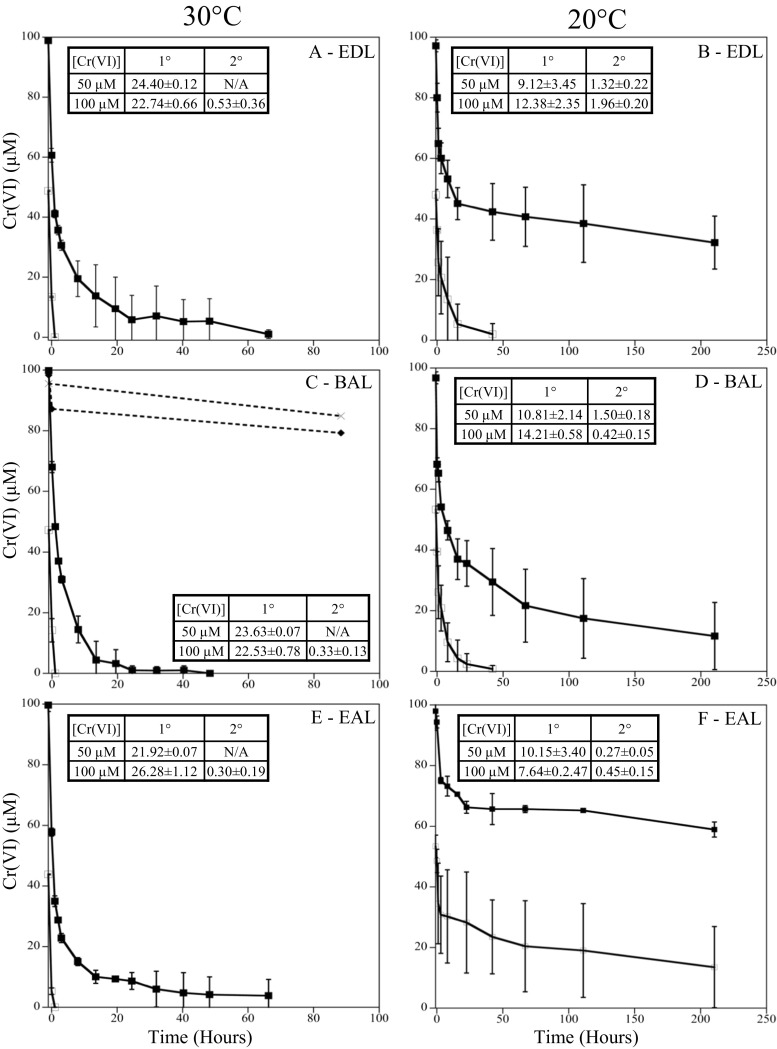
Cr(VI) reduction at 30 °C (**a**, **c**, and **e**) and 20 °C (**b**, **d**, and **f**) under EDL (**a**, **b**), BAL (**c**, **d**), and EAL (**e**, **f**) conditions with 50 (○) and 100 (■) μM Cr(VI). Dashed lines indicate heat-killed (◆) and uninoculated controls (x) (C). Cr(VI) reduction rates (μM Cr(VI)/hr) (primary and secondary) are overlaid for each condition

*D. vulgaris* was grown at both 20 and 30 °C, with 0 and 50 μM Cr(VI), to assess differences in growth rate, biomass yield, and Cr(VI) tolerance at different resource ratios. Under balanced ratio conditions, *D. vulgaris* had a 3.6-fold slower growth rate (0.03 h^−1^) at 20 °C compared to growth at 30 °C (0.12 h^−1^) (Fig. 1c, d). When exposed to Cr(VI) under balanced conditions, *D. vulgaris* could tolerate and reduce Cr(VI) faster at 30 °C compared to at 20 °C. At 30 °C, only a small growth rate difference (10% lower) was observed when cells were exposed to 50 μM Cr(VI) (Fig. 1c, d). At 20 °C and BAL conditions, cells exposed to 50 μM Cr(VI) had an extended lag time (~ 100 h), but the subsequent growth rate was similar to cultures without Cr(VI) (Fig. 1d). In addition, the Y^Sulfate^ values were similar between 0 and 50 μM Cr(VI) once growth was complete. Even under balanced conditions at 20 °C, exposure to 100 μM Cr(VI) caused variable growth and greatly extended lags even though Cr(VI) levels declined (Fig. 2d). For EDL and EAL conditions at 20 °C, 100 μM Cr(VI) caused culture death and incomplete Cr(VI) reduction (Fig. 2). Therefore, growth at 50 μM Cr(VI) was selected as a sub-toxic level to elucidate physiological responses across the tested resource ratios. At 20 and 30 °C, cultures with and without Cr(VI) under balanced conditions utilized lactate and produced equimolar levels of acetate until sulfate was depleted (Fig. S1c). In the presence of 50 μM Cr(VI) under balanced conditions, lactate and sulfate were not utilized during the growth lag, and growth coincided with the utilization of lactate and sulfate (Fig. S1d).

Temperature also affected *D. vulgaris* ability to reduce Cr(VI) (50 or 100 μM) under balanced conditions. At 30 °C, 50 μM Cr(VI) was reduced at a rate of 23.63 ± 0.12 μM Cr(VI)/h whereas at 20 °C, 50 μM Cr(VI) was reduced at an initial rate of 10.81 ± 2.14 μM Cr(VI)/h and a secondary rate of 1.51 ± 0.18 μM Cr(VI)/h (average O.D. 0.08). At 30 °C, 100 μM Cr(VI) was reduced at an initial rate of 22.53 ± 0.78 μM Cr(VI)/h and a secondary rate of 0.33 ± 0.13 μM Cr(VI)/h (average O.D. of 0.08). At 20 °C, 100 μM Cr(VI) was reduced at an initial rate of 14.21 ± 0.58 μM Cr(VI)/h and a secondary rate of 0.42 ± 0.15 μM Cr(VI)/h (average O.D. 0.07) (Fig. 2c, d). Ultimately, a decrease in temperature caused significant decreases in *D. vulgaris* Cr(VI) reduction rates under the balanced condition. Dashed lines refer to abiotic controls with Cr(VI) and medium and C(VI) and heat-killed cells and medium (Fig. 2c).

Previous work has highlighted many different enzymes that are capable of Cr(VI) reduction in *D. vulgaris* (Lovley and Phillips 1994; Chardin et al. 2003; Li and Krumholz 2009), but most studies have been conducted at temperatures selected for maximal growth. Temperature effects on *D. vulgaris* growth and Cr(VI) reduction were pronounced and emphasized the need to study microorganisms at temperatures that are field relevant in order to provide improved estimates for ecosystem function (e.g., Cr(VI) reduction). The difference in Cr(VI) reduction and growth at 20 °C compared to 30 °C is likely due to the combination of a slower growth rate (i.e., slower overall metabolism) at 20 °C and the temperature dependence of enzyme reaction rates. When U(VI) reduction was measured at 20 and 30 °C in a sulfate-reducing consortium, pseudo second-order rate constants for uranium reduction tripled with the 10 °C increase in temperature (Boonchayaanant et al. 2008), further emphasizing the role that temperature plays in microbial processes such as heavy metal reduction. Our results showed that the rate of Cr(VI) reduction during *D. vulgaris* growth was approximately two-fold slower at 20 °C compared to 30 °C and the slower Cr(VI) reduction likely contributed to the increased Cr(VI) toxicity.

### Resource ratio imbalance affects *D. vulgaris* Cr(VI) reduction and tolerance

*D. vulgaris* was grown at both 20 and 30 °C, with 0 and 50 μM Cr(VI) to assess differences in growth rate, biomass yield, and Cr(VI) tolerance under electron-donor limitation. Under EDL conditions, *D. vulgaris* had a 2.9-fold slower growth rate (0.04 h^−1^) at 20 °C compared to growth at 30 °C (0.12 h^−1^) (Fig. 1a, b). When exposed to Cr(VI) under EDL conditions, *D. vulgaris* could tolerate and reduce Cr(VI) faster at 30 °C compared to at 20 °C but to a lesser extent than balanced conditions. At 30 °C and exposure to Cr(VI), the growth rate declined 25% (Fig. 1a). At 20 °C, cells exposed to 50 μM Cr(VI) had an extended lag time that was longer than the balanced condition (~ 125 h), but the subsequent growth rate was similar to the no Cr(VI) treatment (Fig. 1b). At 20 °C, cultures without Cr(VI) under EDL conditions utilized lactate and produced equimolar levels of acetate until lactate was depleted (Fig. S1a). In the presence of 50 μM Cr(VI) under EDL conditions, lactate and sulfate were not utilized during the growth lag, and growth coincided with the utilization of lactate and sulfate (Fig. S1b).

Temperature also affected *D. vulgaris* ability to reduce Cr(VI) under EDL conditions. At 30 °C, 50 μM Cr(VI) was reduced at an initial rate of 24.40 ± 0.12 μM Cr(VI)/h (average O.D. of 0.08) similar to the balanced condition, but at 20 °C, 50 μM Cr(VI) was reduced at an initial rate of 9.12 ± 3.45 μM Cr(VI)/h and a secondary rate of 1.32 ± 0.22 μM Cr(VI)/h (average O.D. of 0.07). At 30 °C, 100 μM Cr(VI) was reduced at an initial rate of 22.74 ± 0.66 μM Cr(VI)/h and a secondary rate of 0.53 ± 0.36 μM Cr(VI)/h (average OD 0.08). At 20 °C, 100 μM Cr(VI) was reduced at a rate of 12.38 ± 2.35 μM Cr(VI)/h that slowed to 1.96 ± 0.20 μM Cr(VI)/h (average O.D. 0.08) (Fig. 2a, b). Ultimately, the decrease in temperature caused a decrease in Cr(VI) reduction rates and the electron donor limitation with 50 μM Cr(VI) caused a 25-h increase in lag time compared to the balanced condition with 50 μM Cr(VI).

*D. vulgaris* was grown at both 20 and 30 °C, with 0 and 50 μM Cr(VI) to assess differences in growth rate, biomass yield, and Cr(VI) tolerance under electron-acceptor limitation. Under EAL conditions, *D. vulgaris* had a 3.5-fold slower growth rate (0.04 h^−1^) at 20 °C compared to growth at 30 °C (0.13 h^−1^) (Fig. 1e, f). When exposed to Cr(VI) under EAL conditions, *D. vulgaris* could tolerate and reduce Cr(VI) faster at 30 °C compared to at 20 °C but to a much lesser extent than balanced conditions. At 30 °C and exposure to Cr(VI), the growth rate declined 24% (Fig. 1e). At 20 °C, cells exposed to 50 μM Cr(VI) had an extended lag time that was longer than the balanced or EDL condition (~ 200 h), and the subsequent growth rate was 15% slower than no Cr(VI) treatment (Fig. 1f). At 20 °C, cultures without Cr(VI) under EAL conditions utilized lactate and produced equimolar levels of acetate until sulfate was depleted (Fig. S1e). In the presence of 50 μM Cr(VI) under EAL conditions, lactate and sulfate were not utilized during the growth lag, and growth coincided with the utilization of lactate and sulfate after 200 h (Fig. S1f).

Temperature also affected the ability of *D. vulgaris* to reduce Cr(VI) under EAL conditions. At 30 °C, 50 μM Cr(VI) was reduced at an approximate rate of 21.92 ± 0.07 μM Cr(VI)/h (average O.D. of 0.08) and at 20 °C 50 μM Cr(VI) was reduced at an initial rate of 10.15 ± 3.40 μM Cr(VI)/h, which then slowed to 0.27 ± 0.05 μM Cr(VI)/h (average O.D. of 0.08). At 30 °C, 100 μM Cr(VI) was reduced at an initial rate of 26.28 ± 1.12 μM Cr(VI)/h that slowed to 0.30 ± 0.19 μM Cr(VI)/h (average OD 0.08) and at 20 °C, 100 μM Cr(VI) was initially reduced at a rate of 7.64 ± 2.47 μM Cr(VI)/h, that slowed significantly to 0.45 ± 0.15 μM Cr(VI)/h (average O.D. 0.07). Ultimately, under the EAL condition and 20 °C, 50 μM Cr(VI) caused an extended growth lag (approximately 200 h) with prolonged exposure to higher Cr(VI) levels compared to the balanced and EDL conditions.

### Viability

To assess if Cr(VI) caused more cell death in EAL grown *D. vulgaris* compared to cells under BAL conditions, the most probable number method was used to compare viability during the lag phases at 20 °C. Viability after 3 h of exposure to Cr(VI) was two orders of magnitude lower under EAL conditions compared to BAL conditions (4.7 × 10^3^ cells/ml vs. 4.7 × 10^5^ cells/ml). At 48 h post-Cr(VI) exposure, the cells under both conditions were starting to recover, but the EAL cells still had decreased viability (2.1 × 10^4^ cells/ml vs. 1.2 × 10^6^ cells/ml). Viability for both EAL and BAL cells was similar under the respective condition without Cr(VI) (approximately 10^8^ cells/ml).

### Growth with increasing sulfate

**Fig. 3:**
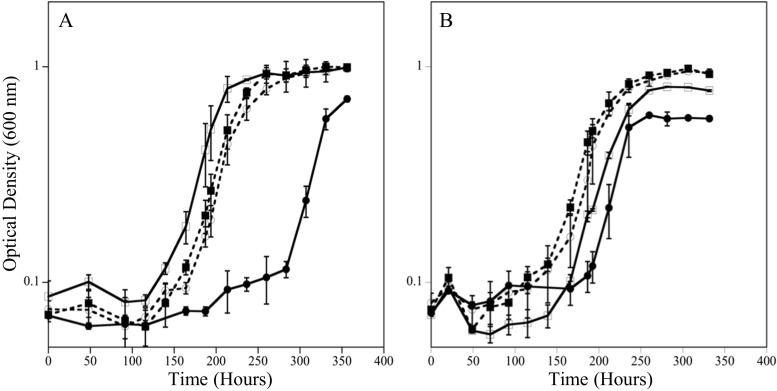
Growth at 20 °C with 50 μM Cr(VI) and increasing sulfate levels of 10 mM (●), 20 mM (◻), 30 mM (■), and 50 mM (○) inoculated with cells grown under EAL conditions (**a**) or BAL conditions (**b**)

To explain the observation that sulfate limitation (EAL condition) resulted in increased Cr(VI) susceptibility, it was hypothesized that chromate competed with sulfate through sulfate permeases/transporters in *D. vulgaris* under the tested growth conditions. Previous work in different organisms has shown that the cellular uptake of chromate oxyanions can occur through sulfate permeases (Pepi and Baldi 1992; Appenroth et al. 2008; Aguilar-Barajas et al. 2011) and chromate was previously shown to block sulfate accumulation and reduction completely in *Desulfovibrio desulfuricans* (Cypionka 1989). To test if increased sulfate levels could alleviate intracellular stress and damage under the EAL condition, cells were inoculated into medium that contained 50 mM lactate, increasing sulfate concentrations (10, 20, 30, and 50 mM sulfate) and 50 μM Cr(VI). In addition, the inoculum cells were either grown in a BAL or EAL condition. Results showed that increasing sulfate concentrations decreased the lag time under both BAL and EAL conditions (Fig. 3). Additionally, inoculum grown under EAL conditions (Fig. 3a) showed an increased lag time when inoculated into EAL medium compared to when inoculum grown under BAL conditions was inoculated into EAL medium (Fig. 3b). These results indicate that the electron donor to acceptor ratio that *D. vulgaris* is grown in prior to Cr(VI) exposure can impact the cellular response in a way that predisposes the cells to increased Cr(VI) susceptibility.

### Growth with normalized sulfate levels

**Fig. 4:**
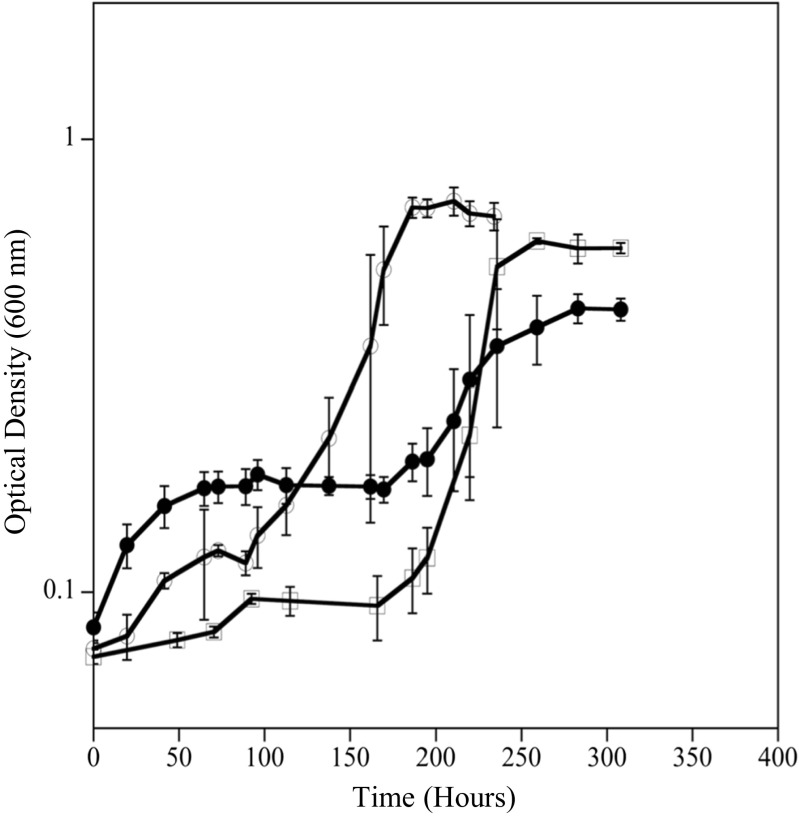
Growth at 20 °C with 50 μM Cr(VI) and lactate to sulfate ratios normalized to 10 mM sulfate. Normalized ratios were EDL (5 mM:10 mM—●), BAL (20 mM:10 mM—○), and EAL (50 mM:10 mM—◻)

Because *D. vulgaris* is the most affected by Cr(VI) under the EAL condition (50:10 mM), Cr(VI) exposure was normalized to nutrient ratios with 10 mM sulfate to determine if increased Cr(VI) susceptibility is caused solely by sulfate concentration or the ratio of lactate to sulfate (BAL—20:10 mM; EDL—5:10 mM, and EAL—50:10 mM). The 20:10 balanced condition lagged for approximately 100 h analogous to the 60:30 mM ratio despite having threefold less sulfate. In addition, the EDL condition (both levels) had a longer lag time than the BAL condition, even when sulfate was 50 mM. (Fig. 4). These results indicate that it is not only the sulfate concentration that affects Cr (VI) toxicity, but also the ratio of electron acceptor to electron donor.

### Sulfate permease mutants

**Fig. 5:**
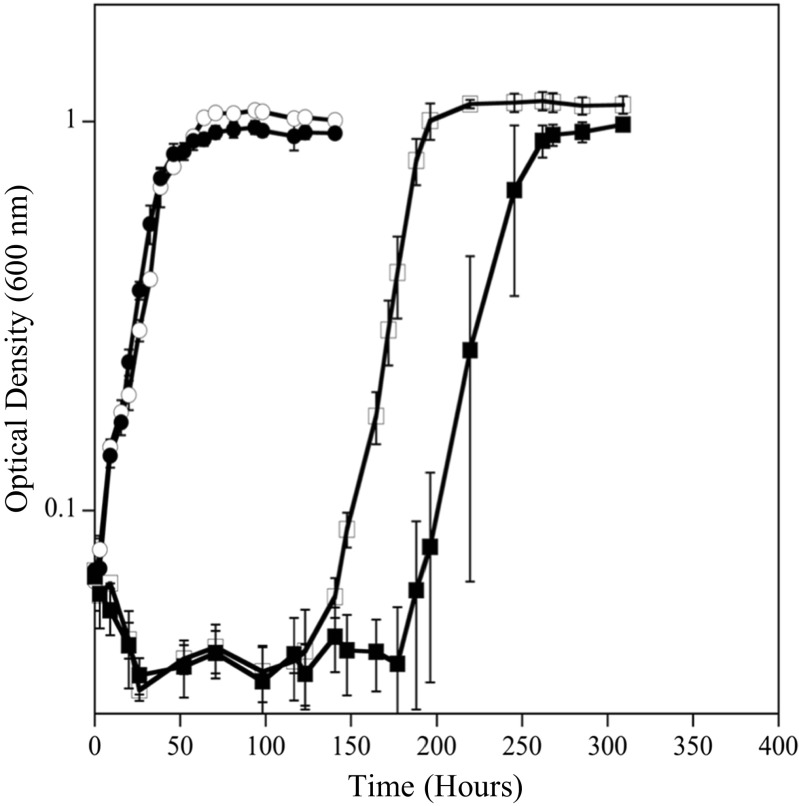
Growth of wild-type *D. vulgaris* (○) and sulfate permease triple mutant (●) without Cr(VI) and with 50 μM Cr(VI) (◻, ■) at 20 °C under BAL conditions

To further understand the role of sulfate permeases in exposure to Cr(VI) in *D. vulgaris*, a search of the annotated genome revealed three genes designated as *sulP* (DVU0053, DVU0279, DVU1999) that are presumptive sulfate permeases in *D. vulgaris* Hildenborough (microbesonline.org). All three presumptive genes are classified in COG659 (sulfate permease and related transporters in the major facilitator superfamily) and annotated to have the SLC26A/SulP transporter domain (IPR011547). Three individual sulfate permease mutants (∆DVU0053, ∆DVU0279, ∆DVU1999) and a triple mutant in which all three sulfate permease genes were knocked out were constructed. The wild-type, three individual mutants, and the triple mutant had similar growth rates and yields when grown via lactate-dependent sulfate respiration without Cr(VI) at 20 °C under BAL conditions (Fig. 5, data not shown for individual mutants). These results indicated that *D. vulgaris* Hildenborough has other mechanisms of sulfate influx (specific and/or non-specific) in addition to the presumptively annotated transporters. When grown in the presence of 50 μM Cr(VI) under BAL conditions, the triple mutant had a longer lag time by approximately 40 h compared to wild-type cells (Fig. 4). These results indicated that the absence of the annotated sulfate permeases did not prevent Cr(VI) from entering and harming the cells and that triple mutant cells had decreased ability to regulate Cr(VI) influx and/or sulfate influx even under balanced conditions. The results suggested that the unidentified mechanism(s) by which Cr(VI) can enter the cell are not as tightly linked to Cr(VI) reduction and/or the alternative mechanisms for sulfate transport have a strong affinity for Cr(VI) that results in increased Cr(VI) sensitivity.

### Ascorbate

**Fig. 6:**
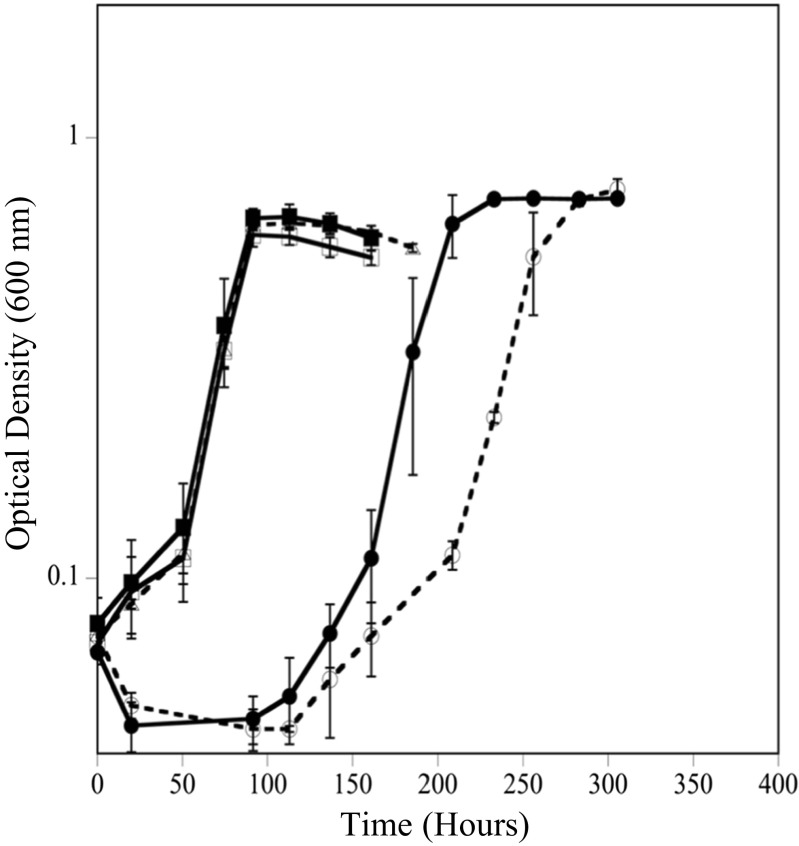
Growth of *D. vulgaris* during Cr(VI) and ascorbate exposure at 20 °C under EAL conditions. Cells were exposed to 50 μM Cr(VI) and 50 μM ascorbate (■), 50 μM Cr(VI) added prior to inoculation and 50 μM ascorbate added at 3 h post-inoculation (●), 50 μM ascorbate (△), 50 μM Cr(VI) (○), or medium only (◻)

*D. vulgaris* was also grown with Cr(VI) in the presence of ascorbate to determine if a reducing/complexing agent could prevent or alleviate Cr(VI) cellular toxicity. Cell growth was compared between cells grown with and without 50 μM Cr(VI), with 50 μM Cr(VI) and 50 μM ascorbate added concurrently, with 50 μM Cr(VI) and 50 μM ascorbate added 3 h post-inoculation, and with 50 μM ascorbate only. When Cr(VI) and ascorbate were added concurrently, *D. vulgaris* cells grew similar to the control without Cr(VI), and these results indicated that the reduced/complexed Cr compounds were not toxic and cells were most likely able to maintain viability (Fig. 6). If ascorbate was added 3 h post-inoculation, the cells were still impacted, but not as drastically as without ascorbate. Approximately 30 μM Cr(VI) remained in solution when the ascorbate was added at the 3-h time point, which might explain why the lag time for this condition was shorter than the condition in which 50 μM Cr(VI) was added without ascorbate.

## Discussion

The mechanism(s) for Cr(VI) reduction in *Desulfovibrio* spp. is not completely understood, but is hypothesized that Cr(VI) enters the cell and is reduced to Cr(III) by cytochrome *c*_*3*_, periplasmic hydrogenases, and possibly thioredoxin reductase (Chardin et al. 2003; Lovley and Phillips 1994; Li and Krumholz 2009). After the Cr(VI) is reduced to Cr(III), the Cr(III) is either excreted from the cell or remains in the inner or outer membrane(s) (Goulhen et al. 2005). It has also been shown that the presence of Cr(VI) alters the metabolism of *Desulfovibrio* spp. by transiently uncoupling lactate oxidation and sulfate reduction and causing lactate to be used for energy production or to lower the redox potential of the medium without concurrent growth at 30 °C (Chardin et al. 2002; Klonowska et al. 2008). The redox potential for Cr(VI) reduction to Cr(III) is 1.41 V (Nriagu and Nieboer 1988); however, *D. vulgaris* Hildenbourough is not known to conserve energy from Cr(VI) reduction and most data indicates that direct and indirect Cr(VI) reduction is linked to detoxification (Chardin et al. 2002; Klonowska et al. 2008). However, microbial fuel cells have recently been shown to reduce Cr(VI) with anode electrons (Habibul et al. 2016), and therefore, improved understanding of electron movement from the bulk phase to heavy metals under different conditions is needed.

Waters contaminated with heavy metals can be deficient in electron donors, electron acceptors, and/or carbon sources related to desired microbial activities, and the exact relationship among growth, activity, and substrate utilization is more challenging to understand when the activity of interest is not directly linked to growth. Previous studies have compared different electron donors ranging from organic acids, alcohols, carbohydrates, and polysaccharides to stimulate Cr(VI) reduction with pure cultures, mixed consortia, and in situ (Liamleam and Annachhatre 2007; Zhang et al. 2015). Geets et al. (2006) tested the removal of Zn, Cd, Co, and Ni from contaminated groundwater in column experiments and concluded that additional experiments were needed to better understand the interplay between sulfate-, metal-, and carbon-source concentrations.

**Fig. 7:**
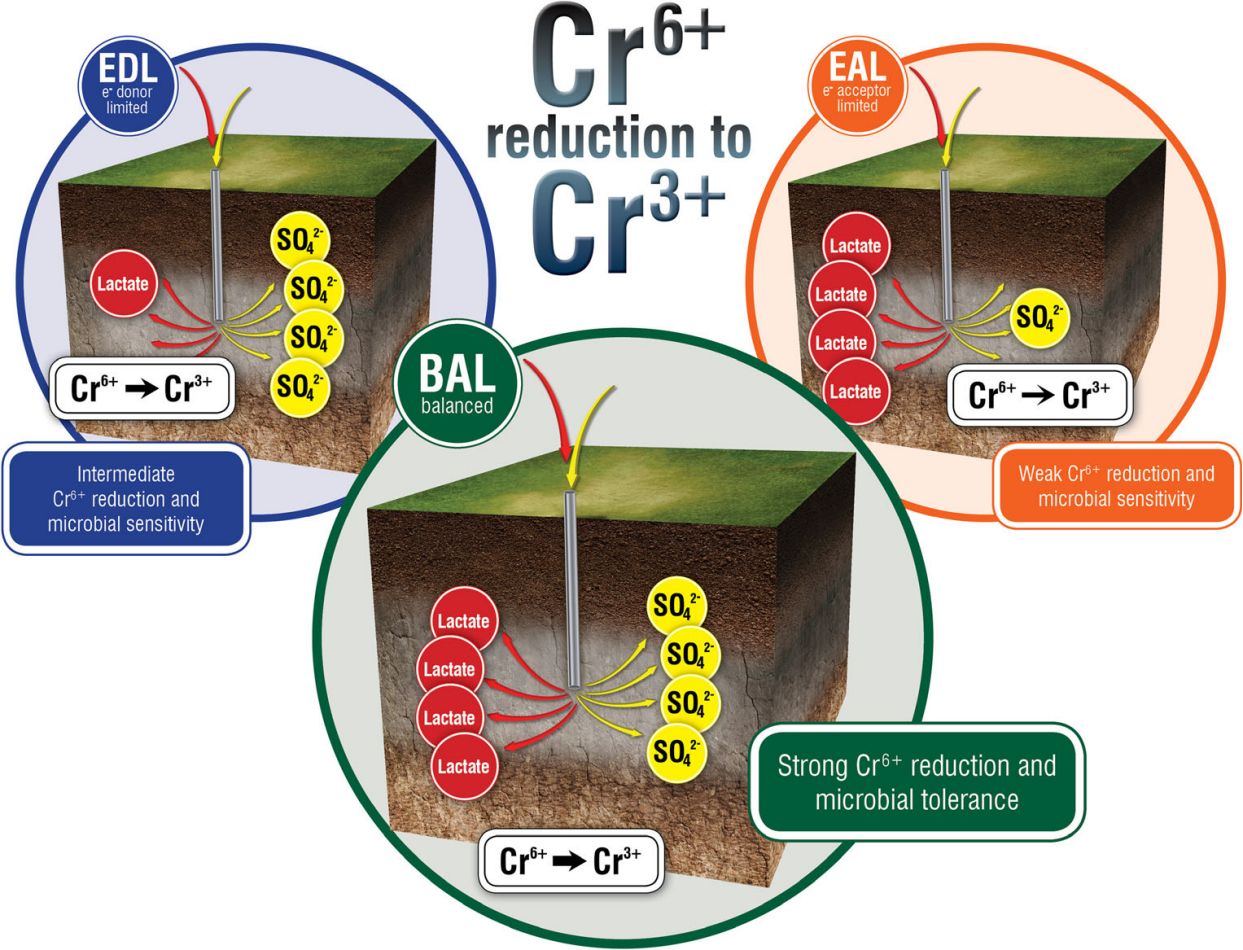
Three potential scenarios (EDL, BAL, EAL) in which nutrients are added to stimulate microbial activity and the ratio of resources are shifted. The different resource ratios can impact desired microbial activity, namely that electron donor/acceptor balanced conditions promote relatively high Cr(VI) reduction and cell viability compared to intermediate Cr(VI) reduction and viability under electron-donor limitation and low Cr(VI) reduction and viability under electron-acceptor limitation

The electron donor to electron acceptor ratio had a very strong effect on Cr(VI) reduction by *D. vulgaris* at both 20 and 30 °C, with the EAL condition at 20 °C being the most detrimentally affected. To explain this, we hypothesized that because chromate (CrO_4_^2−^) and sulfate (SO_4_^2−^) are structural analogues, the amount of sulfate in the environment affects the rate at which sulfate versus chromate enters the cell. It has been shown that Cr(VI) can enter cells through sulfate transport systems in numerous bacteria including, *Pseudomonas fluorescens*, *Salmonella typhimurium*, and *Escherichia coli* (Pardee et al. 1966; Ota et al. 1971; Karbonowska et al. 1977; Ohtake et al. 1987; Sirko et al. 1990), and the slightly longer lag time in the sulfate permease triple mutant in *D. vulgaris* compared to the wild type alludes to the tested permeases playing a role in sulfate and/or Cr(VI) transport. However, the effect does not seem to be merely a sulfate concentration threshold, because the EDL condition has more sulfate compared to the BAL condition (50 vs. 30 mM) and yet the EDL condition has a longer lag time (at 20 °C) compared to the BAL condition. It is important to note that the three resource ratios have similar growth rates without Cr(VI) at 20 and 30 °C. In addition, when the resource ratios were normalized to sulfate level (5:10 mM versus 20:10 mM versus 50:10 for EDL, BAL, and EAL respectively), similar responses to Cr(VI) were observed between EAL and BAL, and EDL had even a longer lag time. These results suggested that the co-metabolic rate of lactate and sulfate utilization influences Cr(VI) influx and reduction, and thus, the subsequent overall cellular toxicity of Cr(VI) and/or Cr(III) (Fig. 7).

To further explore the hypothesis that limiting sulfate increases Cr(VI) toxicity in *D. vulgaris* due to competition between sulfate and chromate for transport across the cell membrane, growth experiments with increasing levels of sulfate and sulfate permease mutants were done. The results of the increasing sulfate experiment support our finding that cells grown under EAL conditions are more susceptible to Cr(VI) toxicity than those grown under BAL conditions. A previous transcriptomic study (Clark et al. 2006) on *D. vulgaris* transitioning from exponential growth to stationary phase shows changes in expression of the annotated sulfate permease genes and other cell components for lactate oxidation as the cells transition from exponential to stationary phase (i.e., decreasing sulfate levels), indicating that perhaps a different cell machinery is used to optimize the utilization of electron donors and acceptors (i.e., presumptive sulfate permeases have different affinities for sulfate). Deletion of the three presumptive sulfate permease genes did not affect growth with lactate and sulfate in the absence of Cr(VI), and this result indicated that sulfate had additional mechanisms to enter the cell. The triple mutant did have an increased lag time when cells were exposed to 50 μM Cr(VI), and this result suggested that the triple mutant had either a decreased affinity/capacity for sulfate influx and/or increased affinity/capacity for chromate influx. An alternative explanation could be an overall decreased metabolic flux due to limited transport, but the triple mutant had similar growth rates in the absence of Cr(VI). Further work is needed to elucidate the additional mechanisms of sulfate transport in *Desulfovibrio* and the role of these transporters in Cr toxicity.

The decrease in biomass yield based on sulfate consumption when cells were exposed to 50 μM Cr(VI) could be explained by the expenditure of energy toward Cr(VI) detoxification and cellular repair from damaging Cr(VI) reduction intermediates instead of allocation toward growth. To determine whether the increased lag time under the EAL condition compared to the BAL condition was due to Cr(VI) causing a greater decrease in cell viability, we measured cell numbers throughout the lag phase in both EAL and BAL conditions with 50 μM Cr(VI). Results indicate that the increased lag time under EAL conditions was due to decreased cell viability, which indicates that Cr(VI) toxicity was more extensive in cells grown in the EAL condition compared to the BAL condition. The interplay between chromate and sulfate levels is most likely a result of a balance between Cr(VI) influx and the ability of cells to reduce Cr(VI) to the less toxic Cr(III), and this detoxification requires adequate reducing potential. The cellular response to Cr(VI) at different resource ratios suggests that *D. vulgaris* modulates metabolic flow in response to the ratio of e^−^/C levels and e^−^ acceptors, and further work is needed to elucidate the mechanisms of metabolic control and the implications for desired activities (e.g., metal reduction). Indeed, previous work has shown that *Desulfovibrio* species do alter activity in response to different electron donors (Lupton et al. 1984), and more recent results showed that intracellular electron flow in *Desulfovibrio* might be modulated respective to e^−^ donor and e^−^ acceptors (Zhou et al. 2017). The increase in Cr(VI) toxicity for sulfate or lactate limited cells has large implications for applications such as bioremediation where carbon and electron sources are injected into the subsurface, most likely creating unbalanced pairings of energy/C sources.

In the subsurface, sulfate-reducing bacteria can account for a majority of biotic metal reduction via direct and indirect mechanisms, but little work has investigated the implications of biostimulation practices on microbial physiology and activity with respect to changing resource ratios and other environmentally relevant conditions (i.e., temperature). The impact of sulfate on Cr(VI) reduction has been previously reported in a yeast and a diatom, and increasing sulfate levels promoted improved efficiencies and rates of Cr(VI) reduction in the yeast and decreased Cr(VI) transport in the diatom (Riedel 1985; de María Guillén-Jiménez et al. 2008). Moreover, the Cr(VI) concentrations that inhibited growth also inhibited sulfate uptake in the diatom (Riedel 1985). Given the role of SRBs in metal interactions in most anaerobic environments (both natural and man-made), further work is needed to ascertain the long-term implications of changing resource ratios on in situ metal exposure, metal-reduction, and metal-toxicity in both monocultures and mixed consortia (Fig. 7).

## Electronic supplementary material

ESM 1 (PDF 7145 kb)
