# Supplementary material for: Cr(VI) reduction and physiological toxicity are impacted by resource ratio in Desulfovibrio vulgaris
Source: Appl Microbiol Biotechnol. 2018 Feb 10;102(6):2839–50. doi: 10.1007/s00253-017-8724-4 (PMC5847207; doi:10.1007/s00253-017-8724-4)
Supplement: Supplementary file 1 — (PDF 7145 kb) [file 253_2017_8724_MOESM1_ESM.pdf]

**Supplemental Materials**

**Applied Microbiology and Biotechnology**

**Title:** **Cr(VI) reduction and physiological toxicity are impacted by resource ratio in *Desulfovibrio vulgaris***

Lauren C. Franco,[a,b] Sadie Steinbeisser,[a,b] Grant M. Zane,[c] Judy D. Wall,[c,d] and Matthew W. Fields[a,b,d*]

Department of Microbiology and Immunology, Montana State University, Bozeman, Montana, USA[a]

Center for Biofilm Engineering, Montana State University, Bozeman, Montana USA[b]

Departments of Biochemistry and Molecular Microbiology and Immunology, University of Missouri-Columbia, Columbia. Missouri, USA[c]

ENIGMA (http:\\enigma.lbl.gov)[d]

*Correspondence: Matthew W. Fields, PhD
366 Barnard Hall
Center for Biofilm Engineering
Montana State University
Bozeman, MT 59717
matthew.fields@biofilm.montana.edu
406-994-7340
406-994-6098

Figure S1

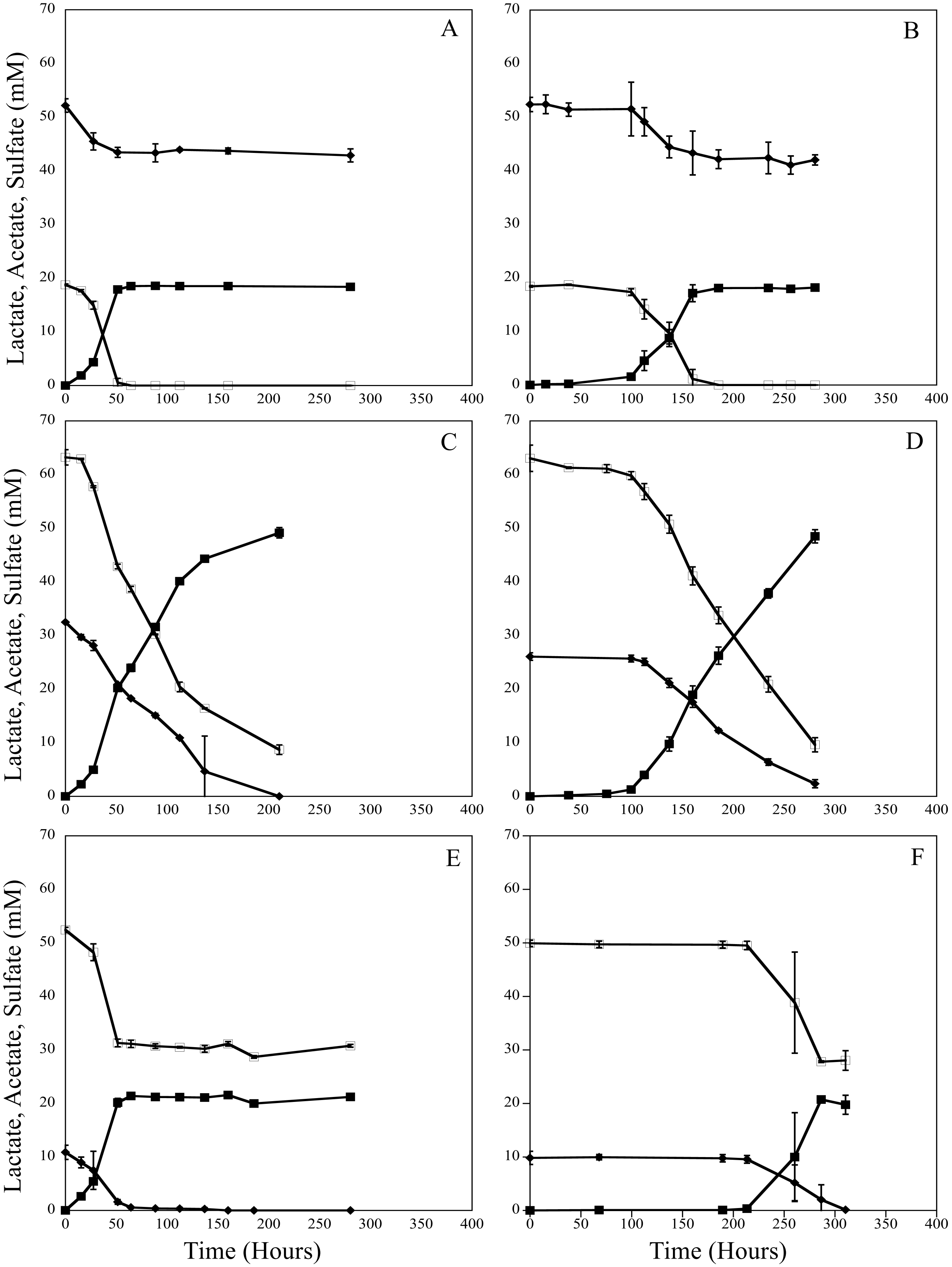


**Figure S1**. Lactate (●), acetate (■), and sulfate (◆) concentrations throughout growth at 20°C with 0 (a, c, e) and 50 (b, d, f) μM Cr(VI) under EDL (a,b), BAL (c,d), and EAL (e,f) conditions.

Table S1. Strains used in this study

| Strain | Features of Interest | Source |
|---|---|---|
| a-select | *deoR endA1 recA1 relA1 gyrA96 hsdR17* ($r_k^-$ $m_k^+$) *supE44 thi-1 phoA* D*(lacZYA argF)U169* F80*lacZ* DM15 l$^-$ F$^-$ | Bioline |
| JW710 | parental strain for deletion strains, D*upp*; 5FU$^r$ | (1) |
| JW9153 | JW710 DDVU0053::($P_{npt}$-*npt-upp*); Km$^r$ 5FU$^s$ | This study |
| JW9155 | JW710 DDVU0279::($P_{npt}$-*npt-upp*); Km$^r$ 5FU$^s$ | This study |
| JW9157 | JW710 DDVU1999::($P_{npt}$-*npt-upp*); Km$^r$ 5FU$^s$ | This study |
| JW9167 | JW9153 DDVU0053 D($P_{npt}$-*npt-upp*); 5FU$^r$ | This study |
| JW9169 | JW9155 DDVU0279 D($P_{npt}$-*npt-upp*); 5FU$^r$ | This study |
| JW9171 | JW9157 DDVU1999 D($P_{npt}$-*npt-upp*); 5FU$^r$ | This study |
| JW9198 | JW9167 DDVU0053 DDVU0279::($P_{npt}$-*npt-upp*); Km$^r$ 5FU$^s$ | This study |
| JW9199 | JW9198 DDVU0053 DDVU0279 D($P_{npt}$-*npt-upp*); 5FU$^r$ | This study |
| JW9200 | JW9199 DDVU0053 DDVU0279 DDVU1999::($P_{npt}$-*npt-upp*); Km$^r$ 5FU$^s$ | This study |
| JW9201 | JW9200 DDVU0053 DDVU0279 DDVU1999 D($P_{npt}$-*npt-upp*); 5FU$^r$ | This study |
| Plasmids | | |
| pCR8/GW/TOPO | parental plasmid, source for pUC Sp$^r$ | Invitrogen |
| pSC27 | source plasmid for $P_{npt}$-*npt*, Km$^r$ | (1) |
| pMO746 | source plasmid for $P_{npt}$-*npt-upp* selectable/counter-selectable cassette; Amp$^r$ Km$^r$ | (2) |
| pMO417 | pCR8/GW/TOPO, marker-exchange plasmid for DVU0053; Sp$^r$ Km$^r$ | This study |
| pMO419 | pCR8/GW/TOPO, marker-exchange plasmid for DVU0279; Sp$^r$ Km$^r$ | This study |
| pMO421 | pCR8/GW/TOPO, marker-exchange plasmid for DVU1999; Sp$^r$ Km$^r$ | This study |
| pMO9152 | pMO417, marker-exchange plasmid for DVU0053, $P_{npt}$-*npt-upp*; Sp$^r$ Km$^r$ | This study |
| pMO9154 | pMO419, marker-exchange plasmid for DVU0279, $P_{npt}$-*npt-upp*; Sp$^r$ Km$^r$ | This study |
| pMO9156 | pMO421, marker-exchange plasmid for DVU1999, $P_{npt}$-*npt-upp*; Sp$^r$ Km$^r$ | This study |
| pMO9166 | pMO417, marker-less deletion plasmid for DVU0053; Sp$^r$ | This study |
| pMO9168 | pMO419, marker-less deletion plasmid for DVU0279; Sp$^r$ | This study |
| pMO9170 | pMO421, marker-less deletion plasmid for DVU1999; Sp$^r$ | This study |
| pMO9154 | pMO419, marker-exchange plasmid for DVU0279, $P_{npt}$-*npt-upp*; Sp$^r$ Km$^r$ | This study |
| pMO9156 | pMO421, marker-exchange plasmid for DVU1999, $P_{npt}$-*npt-upp*; Sp$^r$ Km$^r$ | This study |
| pMO9166 | pMO417, marker-less deletion plasmid for DVU0053; Sp$^r$ | This study |
| pMO9168 | pMO419, marker-less deletion plasmid for DVU0279; Sp$^r$ | This study |
| pMO9170 | pMO421, marker-less deletion plasmid for DVU1999; Sp$^r$ | This study |

Table S2. Primers used in this study

| Name | Sequence* | Purpose |
|---|---|---|
| DVU0053-1b | CGGGAAAGACCTCCGCCTTG | Cloning of upstream region of DVU0053 |
| DVU0053-2 | AAGACTGTAGCCGTACCTCGAATCTA<br>CGTCCCGTGTTCCCTGTTTG | Cloning of upstream region of DVU0053, with overhang for annealing to kanamycin-resistance gene |
| DVU0053-3 | AATCCGCTCACTAAGTTCATAGACCG<br>CATATGGGTGCTGTCAGGTC | Cloning of downstream region of DVU0053, with overhang for annealing to kanamycin-resistance gene |
| DVU0053-4 | AGCAGCCCATGTTGAGGTCG | Cloning of downstream region of DVU0053 |
| bc0050f | TAGATTCGAGGTACGGCTACAGTCTT<br>ATCTCTGAAGAAGCCGACAC<br>CCCCAGAGTCCCGCTCAG | Amplification of kanamycin-resistance gene |
| bc0050r | CGGTCTATGAACTTAGTGAGCGGATT<br>AGTAACAGTCGTGAACATCG<br>GAGGTAGCTTGCAGTGGGCT | Amplification of kanamycin-resistance gene |
| DVU0279-1 | GACTGCGGGAGCATCATGCG | Cloning of upstream region of DVU0279 |
| DVU0279-2 | AAGACTGTAGCCGTACCTCGAATCTA<br>ATGCGCCTCCTTTGCGATTT | Cloning of upstream region of DVU0279, with overhang for annealing to kanamycin-resistance gene |
| DVU0279-3 | AATCCGCTCACTAAGTTCATAGACCG<br>CGTTGATGACAGACGTGACG | Cloning of downstream region of DVU0279, with overhang for annealing to kanamycin-resistance gene |
| DVU0279-4 | ATGAGATTCGCGCCCTGTAC | Cloning of downstream region of DVU0279 |
| bc0051-f | TAGATTCGAGGTACGGCTACAGTCTT<br>ATCGAACTAACGTACATGCC<br>CCCCAGAGTCCCGCTCAG | Amplification of kanamycin-resistance gene |
| bc0051-r | CGGTCTATGAACTTAGTGAGCGGATT<br>ATCGCCTAACCTAGATACAG<br>GAGGTAGCTTGCAGTGGGCT | Amplification of kanamycin-resistance gene |

| | | |
|---|---|---|
| DVU1999-1 | CCCCAAACCCCATCTCGATCGAG | Cloning of upstream region of DVU1999 |
| DVU1999-2 | AAGACTGTAGCCGTACCTCGAATCTA CTGGGGAGACGTTGCGTCTT | Cloning of upstream region of DVU1999, with overhang for annealing to kanamycin-resistance gene |
| DVU1999-3 | AATCCGCTCACTAAGTTCATAGACCG ACCCGACAGTGAGCCGCCAG | Cloning of downstream region of DVU1999, with overhang for annealing to kanamycin-resistance gene |
| DVU1999-4 | ATGATTTGGGCGGCTTCGGC | Cloning of downstream region of DVU1999 |
| bc0052-f | TAGATTCGAGGTACGGCTACAGTCTT GTGTGACATGCTGCTAGAAC CCCCAGAGTCCCGCTCAG | Amplification of kanamycin-resistance gene |
| bc0052-r | CGGTCTATGAACTTAGTGAGCGGATT GCGTCGTAATAGTGGTTATC GAGGTAGCTTGCAGTGGGCT | Amplification of kanamycin-resistance gene |
| pMR->pMLD-Km | GAACACGGCGGCATCAGAG | Amplification of marker-replacement plasmid |
| pMR->pMLD-Cm | GCACCAAGTAAGACTGTAGCCGTACCTCGAATCTA | Amplification of marker-replacement plasmid |
| KanR-upp-pMR-F | AACAGACAATCGGCTGCTCTGATG | Amplification of $kan^r$-upp cassette |
| KanR-upp-pMR-R | TAGATTCGAGGTACGGCTACAGTCTT ACTTGGTGCCGAATATCTTGTCGCC | Amplification of $kan^r$-upp cassette; contains overhang for common sequence |
| DVU0053-MLD-F | GGAACACGGGACGCATATGGGTGCTGTCAGGTCTTCG | Amplification from marker-replacement plasmid to construct the marker-less deletion plasmid for DVU0053 |
| DVU0053-MLD-R | CAGCACCCATATG CGTCCCGTGTTCCCTGTTTGC | Amplification from marker-replacement plasmid to construct the marker-less deletion plasmid for DVU0053 |
| DVU0279-MLD-F | AAAGGAGGCGCATCGTTGATGACAGACGTGACGTTCC | Amplification from marker-replacement plasmid to |

| | | |
|---|---|---|
| DVU1999-MLD-R | CTCACTGTCGGGT CTGGGGAGACGTTGCGTCTT | Amplification from marker-replacement plasmid to construct the marker-less deletion plasmid for DVU1999 |
| | | |

* - underlined region represents overhang used for annealing to neighboring PCR product in SOE and SLIC reactions.

REFERENCES


1. **Keller KL, Bender KS, Wall JD.** 2009. Development of a markerless genetic exchange system for *Desulfovibrio vulgaris* Hildenborough and its use in generating a strain with increased transformation efficiency. Appl Environ Microbiol **75**:7682–7691.
2. **Parks JM, Johs A, Podar M, Bridou R, Hurt RA Jr, Smith SD, Tomanicek SJ, Qian Y, Brown SD, Brandt CC, Palumbo AV, Smith JC, Wall JD, Elias DA, Liang L.** 2013. The genetic basis for bacterial mercury methylation. Science **339**:1332–1335.
